# Soft Grippers for Automatic Crop Harvesting: A Review

**DOI:** 10.3390/s21082689

**Published:** 2021-04-11

**Authors:** Eduardo Navas, Roemi Fernández, Delia Sepúlveda, Manuel Armada, Pablo Gonzalez-de-Santos

**Affiliations:** Centre for Automation and Robotics, UPM-CSIC, Carretera CAMPO-REAL Km 0.2, Arganda del Rey, 28500 Madrid, Spain; delia.sepulveda@csic.es (D.S.); manuel.armada@csic.es (M.A.); pablo.gonzalez@car.upm-csic.es (P.G.-d.-S.)

**Keywords:** soft robotics, agriculture 4.0, soft grippers, end-effectors, review, harvesting process

## Abstract

Agriculture 4.0 is transforming farming livelihoods thanks to the development and adoption of technologies such as artificial intelligence, the Internet of Things and robotics, traditionally used in other productive sectors. Soft robotics and soft grippers in particular are promising approaches to lead to new solutions in this field due to the need to meet hygiene and manipulation requirements in unstructured environments and in operation with delicate products. This review aims to provide an in-depth look at soft end-effectors for agricultural applications, with a special emphasis on robotic harvesting. To that end, the current state of automatic picking tasks for several crops is analysed, identifying which of them lack automatic solutions, and which methods are commonly used based on the botanical characteristics of the fruits. The latest advances in the design and implementation of soft grippers are also presented and discussed, studying the properties of their materials, their manufacturing processes, the gripping technologies and the proposed control methods. Finally, the challenges that have to be overcome to boost its definitive implementation in the real world are highlighted. Therefore, this review intends to serve as a guide for those researchers working in the field of soft robotics for Agriculture 4.0, and more specifically, in the design of soft grippers for fruit harvesting robots.

## 1. Introduction

In the last decade, the agricultural sector has undergone a deep transformation to cope with the growing demand for food [1,2,3]. Among the main tasks in agricultural processes, those that involve the manipulation of fruits and vegetables continue to be one of the most time consuming and labour intensive, resulting in low efficiency and limited competitiveness. This situation is exacerbated by the labour shortages of seasonal workers unable to travel between regions, leading to the accumulation of fresh products and impressive food losses. For these reasons, a great research effort is underway to automate these manual operations, as in the case of selective harvesting, combining multidisciplinary fields such as biological science, control engineering, robotics and artificial intelligence. Special emphasis is being placed on topics such as the modification of plant peduncles [4], which could simplify the harvesting process [5]; machine vision and detection systems [6,7,8,9,10]; decision-making architectures [11,12,13]; autonomous navigation [14,15,16]; and dexterous manipulation [17,18]. Another critical topic, often underestimated, is that related to the design of the systems attached to the tip of robotic manipulators and that are in direct contact with the fruit, known as grippers or end-effectors.

In manual harvesting, humans use their hands to move different elements of plants, grasp the fruits and detach them, either directly or with the help of a tool. The kinematics of human hands, the deformability of the skin and muscle, and their sense of touch give us efficient grasping abilities. Attempts to emulate human skills during harvesting have resulted in numerous mechanical end-effectors that can be classified according to their numbers of fingers into two major groups: multi-fingered and parallel grippers [19].

Multi-fingered grippers, such as those proposed in [20,21,22,23], include multiple degrees of freedom (DoFs), giving them grasping characteristics similar to the human hand, although they are expensive and difficult to control due to the large number of actuators. On the other hand, parallel grippers exhibit a simpler mechanical structure, making them easier to control, as they have fewer actuators. However, this simplification translates into less adaptability during grasping.

With the emergence of soft robotics, grippers based on soft and deformable materials have recently begun to be proposed for industrial and medical applications [24,25,26,27,28,29,30,31]. These soft grippers, which are able to continuously vary their shape without requiring complex multi-joint mechanisms, have the potential to provide greater adaptability while presenting lower costs and simpler structures and control algorithms than hard end-effectors [32,33].

With all this in mind, this review aims to present and discuss the latest developments in the design and implementation of novel soft grippers and end-effectors. To that end, the suitability of each of the proposed grippers to the movements required during the harvesting processes is studied, as well as their manufacturing processes and low-level control methods.In addition, the picking patterns (i.e., movements required to harvest the fruit) reported in the literature are analysed, and classification is presented in which the correct picking patterns for a considerable number of fruits are identified. Moreover, a list of the remaining challenges for the implementation of soft grippers in robotic crop harvesting is presented.

Therefore, the beneficiaries of this review can be all companies, designers or researchers who want to see a complete picture of the current progress of soft robotics and its suitability for implementation in the agricultural sector. The rest of the review is organized as follows. In Section 2, an overview of the current state of robotic harvesting automation is introduced, delving into the most critical aspects for gripper design, such as the characteristics of the picking patterns and the nature of the different fruits. Section 3 describes the soft technologies applied to existing grippers that could be used for Agriculture 4.0 applications, as well as the main control solutions implemented for soft grippers. Section 4 lists the main challenges of soft grippers for robotic crop harvesting. Finally, Section 5 summarizes the major conclusions.

## 2. Harvesting Process

### 2.1. Harvesting Process Classification

Since gripper designs for robotic harvesting are highly dependent on the picking process, the main techniques currently in use are summarized below with the aim of finding the gaps where soft robotics can make the greatest contributions. The general classification presented in [34] divides the detachment of fruits into two methods: (i) mechanical detachment, which involves the removal of pieces of fruit from the tree branch by means of a machine or a mechanical mechanism, and (ii) manual detachment, which consists of the extraction of pieces of fruit from the tree branch by the human hand. In [35], mechanical fruit harvesting processes are classified as follows: (i) those that remove the fruits by shaking the entire plant through air blasting, canopy shaking, limb shaking or trunk shaking; sometimes these methods are assisted with a chemical agent, which makes ripe fruits easier to harvest; and (ii) those that use automatic robotic picking machines that require minimal or no human intervention in their operation.

**Figure 1:**
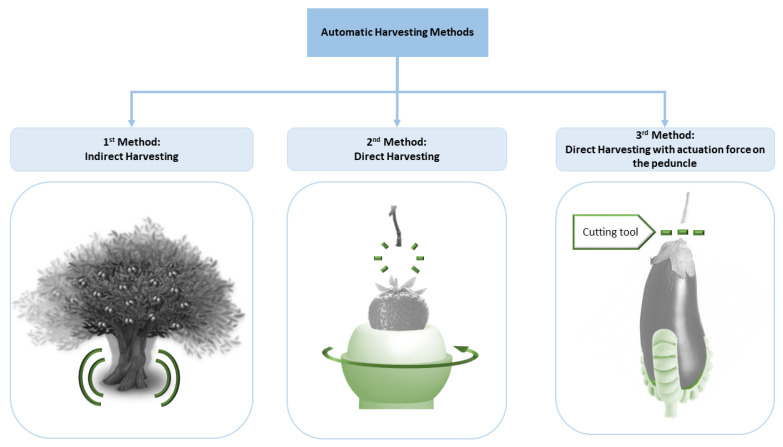
Classification of automatic harvesting methods.

With the introduction of a wide variety of robotic solutions for fruit harvesting and the design of new grippers and end-effectors in recent years, it is convenient to update the classification of automatic harvesting methods to include these latest technologies. The classification proposed in this review is an extension of that carried out in [36], which classified the removal of the fruits into two groups: (i) those in which the application of direct force to the harvested portion is necessary and (ii) those that deliver the removal energy indirectly as an inertial force response that causes detachment by accelerating the attachment support away from the harvest object. Consequently, harvesting methods are divided into three main groups, which are shown schematically in Figure 1:

(1) Indirect harvesting: a technique that involves indirect mechanical movement towards the fruit through a force applied to the plant itself, such as that carried out when harvesting olives [37], almonds [38] or pistachio nuts [39]. To make the fruits fall without any contact points, methods such as air blasting, limb shaking, trunk shaking and canopy shaking are often used [34,35].
(2) Direct harvesting: a method used in those crops that, due to the structural characteristics of the plant, cannot be shaken but require the direct application of a mechanical force on the fruit or its peduncle; these picking techniques, which are discussed in more detail in Section 2.2, are also known as picking patterns (e.g., twisting, pulling or bending) and cause fruits to detach from the stem [40]. Examples from this group are the methods used in the harvesting of strawberries [40,41], apples [42,43,44,45] and several varieties of tomatoes [46,47,48,49].
(3) Direct harvesting with an actuation force on the peduncle: a technique that is applied to those fruits that require a direct mechanical movement, or another type of cutting method, applied directly to the stalk since due to their morphology they are connected to the plant by a hard peduncle that must be cut, as in the harvest of aubergines [50,51], melons [52], oranges [53], cucumbers [54] and peppers [55,56,57].

In the classification of the harvesting processes presented above, it is important to highlight that the fruits included in the first group can also be harvested using the methods described in the second group due to the physical characteristics of the peduncle. The most suitable harvesting method to use must be studied on an individual basis depending on the crop. Several factors may influence the choice of the most suitable harvesting method, such as (i) the size and shape of the tree [36], (ii) the structural fragility of the plant [35,36], (iii) the maturity stage of the fruits [34,58], (iv) the lack of preharvesting chemical fruit looseners, which affect the ease of harvesting [34], (v) the requirements of avoiding damage to the fruit or the plant [36,58] and (vi) the financial profitability [34]. Some authors [34,59] discourage the use of products such as chemical fruit looseners before harvest due to their effect on the defoliation of the trees and the subsequent lack of bloom in the following year. This complicates harvesting through indirect contact of the various fruits within the first group, which in some cases are collected by air blasting, limb shaking, trunk shaking or canopy shaking [34].

There are also several differences between the requirements of the group 2 and group 3 techniques. For instance, the harvesting methods included in group 3 need a more sophisticated perception system than those in group 2, since in addition to the fruits, they have to detect peduncles; they also require a robotic system with greater precision to locate the peduncle between the blades of the tool and proceed to cut it without damaging the crop [47], while with the group 2 techniques, fruits can be harvested with part of the peduncle with just one picking action.

In the literature, authors of [60] resume the main capabilities of an ideal picking robot as the following: (i) the 3D location of the fruits in the plant, (ii) trajectory planning, (iii) the application of detachment method and adequate storage, and (iv) the application of reliable driving system. These operations must be carried out under the constraints of (i) increasing the harvest ratio between robotic picking and manual picking, (ii) increasing the quality of the harvested fruit, and (iii) being economically justified. Furthermore, ref. [61] highlights two main challenges in fruit harvesting: (i) an adequate manipulation of fruits to avoid the loss of quality and consequently, the loss of value in the market, which implies the development of grippers and end-effectors that meet this requirement, and (ii) the study of the detachment method for removing the fruit from the tree, which varies according to the type of fruit.

### 2.2. Picking Patterns

**Figure 2:**
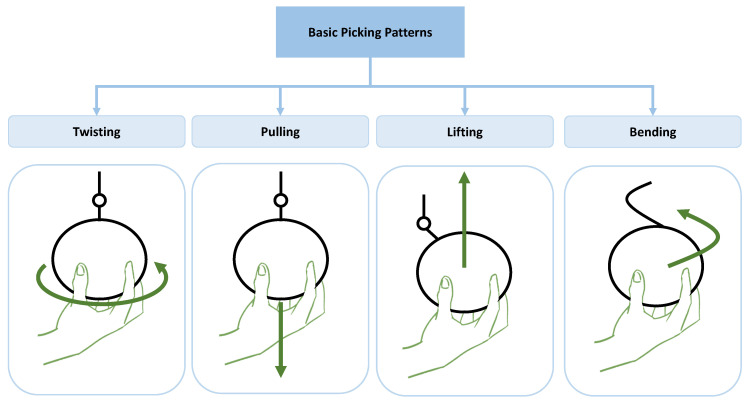
Simplified scheme of basic picking techniques.

As stated above, the fruits harvested by means of the methods classified in group 2 pose a challenge in the field of robotic manipulation. One of the research paths in this field is based on the idea of studying and decomposing the human movements performed during the harvesting of fruits and replicating them using robotic grippers. These movements are grouped under the concept of picking patterns, which include, among others, the movements of bending, lifting, twisting, and pulling or a combination of them. In Figure 2, the basic picking patterns are shown conceptually.

An important factor that has been studied within the field of biological science for group 2 methods, and particularly for the application of the picking patterns, is the abscission layer, which is a barrier of thin-walled parenchyma cells that develops between the fruit and the fruit stalk or the fruit stalk and the branch. This development process occurs when the moment of the fall of a fruit from a plant approaches to facilitate detachment [62]. In most cases, fruit harvested before development of the abscission zone will not have well-developed sugar, volatile, or flavour attributes [63]. Some investigations are trying to modify or eliminate this layer by modifying the plant so that the next point of separation of the plant from the fruit is located right in the calyx and the fruit is easier to harvest [5,62]. Therefore, the identification of the abscission layer is important to determine where the fruit separates from the plant at the time of harvest, as well as the picking patterns to apply.

**Figure 3:**
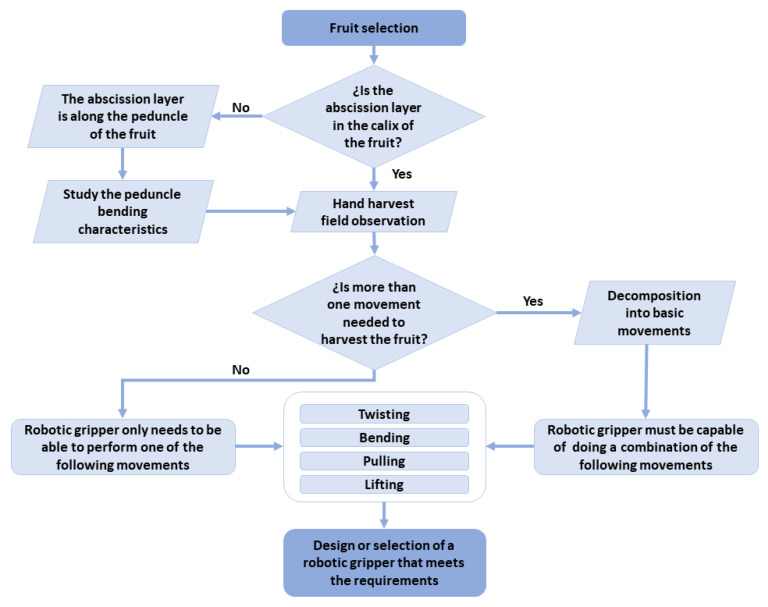
Steps to design or select a gripper or end-effector based on the study of a picking pattern.

In the literature, there are studies available on picking patterns for (i) tomatoes [46,47,64], (ii) kiwis [65], (iii) apples [61] and (iv) strawberries [66,67]. It is also worth mentioning the study presented in [68], in which the movements of the hand and the human body in the harvesting process are analysed to provide a guide for the design of new grippers and end-effectors of anthropomorphic inspiration. The scheme shown in Figure 3 summarizes the proposed steps to follow for the design or selection of grippers and end-effectors required to harvest fruits by means of direct contact methods.

Since the picking patterns described in this section involve direct contact with the fruit, the introduction of soft grippers may represent a significant advance in the automation of the harvesting methods classified in group 2, allowing a delicate manipulation that guarantees the integrity of fruits.

### 2.3. Direct Harvesting with an Actuation Force on the Peduncle

Regarding group 3, a comprehensive classification of the types of mechanisms used in grippers coupled to manipulators for the harvesting techniques of group 3 can be found in [2,24]. Research studies on this third group [69,70,71,72,73,74,75] have focused on the shear characteristics of the plants, such as the shear ultimate stress, the maximum force and the shear energy. These characteristics could be helpful in the study of the peduncles of fruits, with the aim of developing more energy-efficient cutting tools. For cutting peduncles, there are several techniques that can be classified into two groups: (i) techniques based on the bending characteristics of the stalk, such as the bending force, bending stress and Young’s modulus, and (ii) techniques based on the shear characteristics, such as the shear force, shear strength and shear energy. Table 1 presents the classification of several cutting techniques. According to this table, the tools that do not use the bending force have in common the need to consider the cutting characteristics, in particular, the cutting force and the cutting energy required to separate the fruit from the plant. Since laser cutting is not based on the peduncle characteristics, it has not been included in this table [76].

Therefore, harvesting techniques of group 3 are also candidates for the introduction of soft gripper technology, provided they are complemented by a suitable cutting tool.

### 2.4. Literature Overview of Crop Harvesting Automation

Table 2 and Table 3 present a collection of articles that propose technological solutions for automatic harvesting, botanically classified according to the target fruit [80]. This botanical-based classification divides fruits into simple fleshy, aggregate and multiple. Simple fleshy fruits (such as a berries, drupes, or pomes) are those derived from a single ovary of and individual flower [81]. Aggregate fruits (such as raspberries) consist of many individual small fruits derived from separate ovaries within a single flower, borne together on a common receptacle [82]. Lastly, multiple fruits (such as figs, mulberries, or pineapples) are those derived from the ovaries of several flowers that coalesce into a single structure [82].

In addition, in each table, the harvesting method used is identified, following the classification of harvesting techniques proposed above and taking as a reference both the information presented in [36,83] and the visualization of the harvesting processes. Although the proposed solutions may be valid for several crops, they have been assigned only to those crops where an experimental study has been reported. Additionally, it is taken into account that the crops classified in groups 2 and 3 are the most suitable for the adoption of soft gripper technology.

## 3. Soft Grippers

Soft grippers are those end-effectors that use materials and actuation methods that are soft, flexible and compliant and that enable the holding of an object to be manipulated. The softness characteristic provides the adaptability and robustness seen in natural organisms, allowing grasping and manipulation to be achieved with ease. These systems have the potential to interact more safely within an unstructured human environment and deal with dynamic and uncertain tasks [182].

Since fruits must be handled properly to avoid the loss of quality and reach their maximum value in the market, soft grippers are presented as one of the best solutions for harvesting crops, given their adaptability and the delicacy with which they can grasp and manipulate the target products.

**Figure 4:**
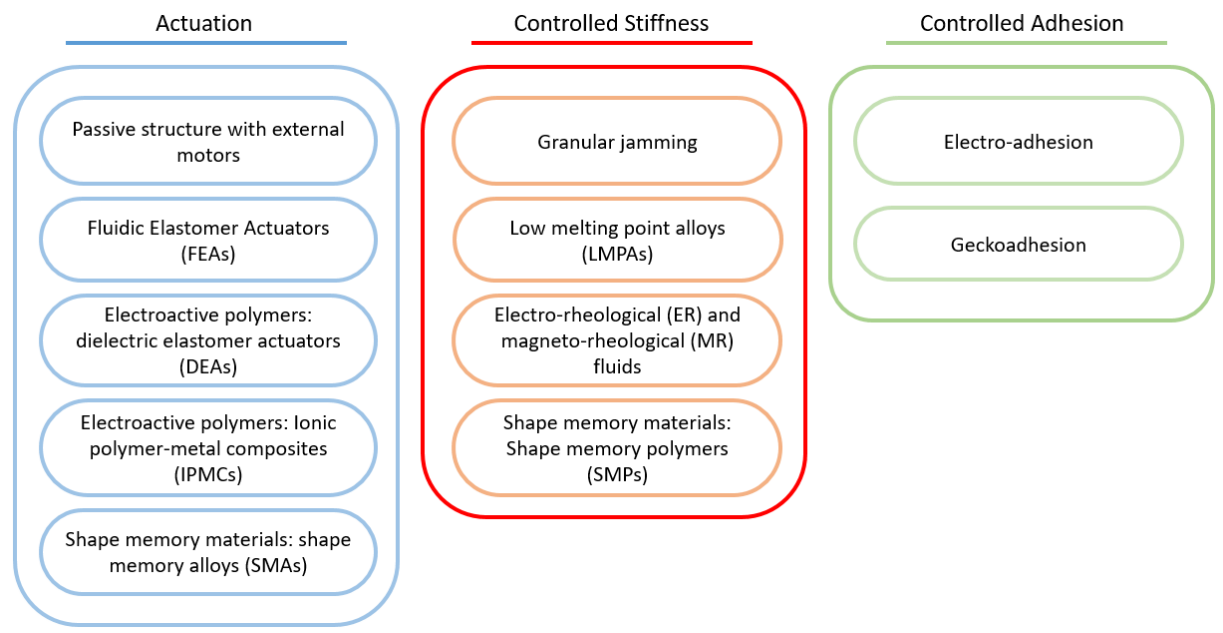
Classification of soft gripping technologies proposed by [190].

In this context, soft technologies can be defined as the set of theories, techniques and procedures that enable key functions of soft robotic grippers, such as actuation, gripping and shape control methods. Although different authors have proposed a great variety of soft technologies [183,184,185,186,187], the main objective of all of them is to guarantee the safe interaction of the device with humans and the environment by using materials with a module similar, in terms of rigidity, to that of soft biological materials [187]. Several reviews of soft grippers can be found in the literature [19,33,182,183,184,187,188,189], presenting various approaches to classify existing technologies. One of the most widely used approaches is the one that classifies the soft gripping technologies according to three different categories [190,191]: (i) actuation, (ii) control stiffness and (iii) controlled adhesion. However, it is currently possible to find devices whose designs simultaneously combine characteristics from several of these categories. Figure 4 shows the complete classification of the current soft gripping technologies based on the mentioned categories.

From an agricultural point of view, some of these technologies may be more relevant than others. Based on the reviews carried out in [189,190], evaluation criteria adapted to Agriculture 4.0 can be established to perform a quantitative and qualitative analysis of the existing soft grippers. These criteria are listed below.

- Object size: This is one of the most critical aspects to evaluate soft technology since its use in certain crops depends on it. Passive structures with external motors, fluidic elastomer actuators (FEAs) and controlled adhesion are the technologies with the best capacity to grasp large objects.
- Gripper size: Another criterion is the size of the device, which can be critical to access certain crops.
- Lifting ratio or operation range: This variable can be interpreted as the ratio between the mass of the object and the mass of the gripper or as the force that the soft actuator can exert. If interpreted as a ratio, it should always be related to the maximum size of the object that can be grasped. For example, shape memory alloy (SMA) actuators have a higher lift ratio than FEAs but a less manipulable object size, which reduces their suitability for fruit picking.
- Power consumption: Each soft technology requires a different type of support device. The technologies that require electric motors or pumps to operate demand the highest energy consumption.
- Scalability: This feature takes into account not only the ease of manufacture but also the modularity of the technology used. This is especially important for the adaptation of soft grippers to various types of crops, and it is desirable that they be as universal as possible to increase their viability.
- Controllability: Depending on the soft technology used, several proposals for low-level control systems can be found. Normally, the most widely used control method is open-loop. With respect to fluidic actuators, liquid-based devices can exhibit more linearity than pneumatic devices.
- Response time: This variable can affect the efficiency of the agricultural task. It may be difficult for soft actuators that rely on a fluid to achieve high actuation frequencies due to the fluidic impedance of the channel and the flow actuation level.
- Surface conditions: Soft gripper technologies that require a clean surface, such as controlled adhesion, are less suitable than those that do not have any surface-related requirements.
- Degree of skill to working in unstructured environments: Although soft technology is one of the most suitable for working in unstructured environments, not all soft grippers that can be found in the literature are suitable for agriculture scenarios. This is the case for devices that require complex support devices that are sensitive to large holes or that can suffer tears from sharp objects [192].
- Mechanical compliance: Each soft technology has an advantage in terms of compliance. For instance, FEAs, shape memory polymer (SMP) actuators and dielectric electroactive polymer (DEAP) actuators are inherently compliant due to the materials used. With other technologies, such as SMA actuators, this parameter depends on the shape of their structures.
- Lifetime: The parameter is the number of cycles that a soft actuator can remain in operation before failing or exhibiting altered motion patterns. Lifetime is an important characteristic in FEA technology, which is subjected to constant fill and empty cycles that tend to wear away the material.
- Technology readiness level (TRL) [189]: Another criterion to compare the feasibility of each technology could be the TRL. Those that have experimentally demonstrated their efficiency in real operating environments, as well as those that are also easier to put into production due to the type of support devices they use and the materials and manufacturing process they require, have a higher TRL.

According to this classification, controlled adhesion technology may be difficult to adapt to agricultural tasks, as it requires a special surface to be able to grip an object, although the weight lifted/weight gripper ratio (39 [193]–286.7 [194]) and the size of the object could be suitable ($0.16\times 10^{-2}$ m [195] to $100\times 10^{-2}$ m [196]). Regarding the grippers grouped around control stiffness, granular jamming ones stand out, since they have a good weight lifted/weight gripper ratio, as well as a good response time and the ability to lift small to medium-size fruits. The other components in this group are discarded for harvest purposes since their performance is not ideal for these tasks. Finally, in the actuation technology group, passive structures with external motors and FEA actuators could be ideal ones for fruit harvesting grippers because (i) they have a large lifted size/gripper weight ratio; (ii) the size of the object can be between 0.01 and 100 × $10^{-2}\;m$, which includes the sizes of most fruits; (iii) they have a good response time; and (iv) they have the ability to grasp any object. A disadvantage may be their energy consumption since they are hampered by the need for an electric motor or pump. Nevertheless, these technologies present the highest TRL level, which would facilitate their production.

### 3.1. Materials and Manufacturing Methods

#### 3.1.1. Materials

**Figure 5:**
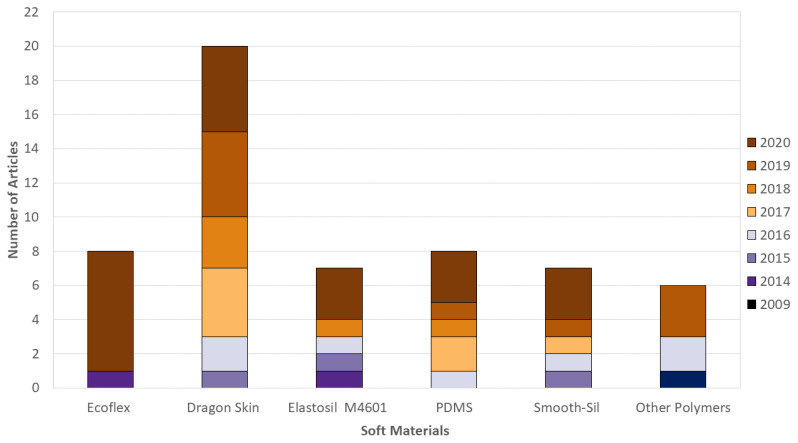
Silicone elastomers and other polymers used in soft robotics literature, as well as the corresponding number of citations. For this graph, 45 articles were examined: Ecoflex [198,199,200,201,202,203,204,205], Dragon Skin 10/20/30/FX-Pro [206,207,208,209,210,211,212,213,214,215,216,217,218,219,220,221,222,223,224,225], Elastosil M4601 [199,202,218,226,227,228,229], PDMS [198,208,230,231,232,233,234,234], Smooth-Sil [200,207,209,218,222,223,227] and Other Polymers [235,236,237,238,239,240,241].

As mentioned above, a wide variety of soft grippers have been proposed. Soft components typically used in the actuators of these grippers include urethanes, hydrogels, braided fabrics, hydraulic fluidics and polymers, such as silicone elastomers [197]. However, actuators based on silicone elastomers have attracted strong interest due to their low cost and ease of manufacture; they do not require the use of complex machinery or skilled labour. In addition, these compliant materials are also advantageous when considering the safety of interaction with biological products, making them appropriate candidates for agricultural applications. Figure 5 presents a bar graph showing the commercially available materials (silicone elastomers and other polymers) that are most frequently reported in the soft robotics literature and that consequently can be used for soft grippers implementation.

Several of these soft materials, particularly silicone elastomers, can be modelled as rubber elastomeric membranes that are hyperelastic and nearly incompressible. Various approaches based on developing free energy density functions can be found to describe the phenomenological constitutive models of rubber-like materials, such as the Neo-Hookean, Mooney–Rivlin (Mooney, 1940; Rivlin, 1948), Ogden (Ogden et al., 2004) and Gent models (Gent, 1996).

As shown in Figure 5, the five most commonly used materials are Dragon Skin, Ecoflex, polydimethylsiloxane (PDMS), Elastosil M4601 and Smooth-Sil, which are all silicone elastomers. Other polymers are Agilus30/VeroClear, ultra-high molecular weight polyethylene, electrostatic discharge (ESD) plastic sheet, thermoplastic elastomers (TPEs) and thermoplastic polyurethane (TPU).

Although there are no specific studies that categorically confirm the suitability of the above materials for the agricultural sector, all materials are declared in their safety data sheet as non-hazardous substances. However, it would be convenient to carry out studies that analyse the life cycle of soft actuators made with these materials, to determine if their degradation may leave particles on the products manipulated.

Dragon Skin, Ecoflex and Smooth-Sil are commonly used for manufacturing objects outside the scientific field, so determining their exact chemical composition is difficult. However, they are versatile, easy to use and handle, and low cost compared to other silicones, and their hardness is between 10 and 50 Shore A. Elastosil M4601 is highly resistant to bending and elongation; it has low viscosity in its uncured form, which makes it easy to mould; and its hardness is approximately 28 Shore A. PDMS has high elasticity [242], it is a thermoset [230], and its behaviour can be mathematically modelled with great precision by means of finite element method (FEM) analysis due to its well-known chemical composition. Furthermore, the variation in its hardness through several mixing ratios has been extensively studied in the literature [243,244]. The main advantage of other soft materials, such as TPU and TPE, is that they can be 3D printed. Additionally, another advantage of TPU-95 is its durability (85A Shore hardness), making it suitable for agricultural environments, where harmful collisions with objects are frequent [236].

A common advantage of all of these silicones is their ability to cure at room temperature, without the need for an oven, although an oven can be used to shorten the cure time.

#### 3.1.2. Manufacturing Methods

The main soft actuator manufacturing methods, comprehensively reviewed in [182], are (i) the moulding process, where fused deposition modelling (FDM) printers are commonly used for mould making; (ii) shape deposition manufacturing (SDM), which facilitates the construction of 3D soft actuators composed of multiple materials with different properties; (iii) soft lithography, which facilitates the development of multichannel soft actuators; (iv) lost-wax cast fabrication [245]; and (v) soft 3-D printing. The latter can be considered a promising technology due to the elimination of several moulding stages, which facilitates the manufacturing process and the design of more complex inner chambers or pneumatic networks.

### 3.2. Soft Grippers for Food

In the field of soft robotics, particularly in soft grippers, there is a lack of soft actuators designed for picking fruits and vegetables. This absence is most noticeable for harvesting tasks. Although this is discussed in more detail in the following sections, note that the handling of this type of product requires precise control of the gripper to successfully carry out the movements of the picking patterns that are listed above without damaging the fruit. Furthermore, the current state-of-the-art soft actuators tend to be researched in the field of manipulation, which in many cases is very generalist and is not particular to the diverse characteristics of individual objects.

However, in the field of industrial food handling, there are more research studies that could be considered the basis for soft grippers in Agriculture 4.0 applications. These studies are listed below, classified according to the type of soft actuator they use, indicating the advantages of each technology. Only studies that specifically refer to food handling have been taken into account.

- FEA [206,220,224,225,230,236,246,247,248,249,250,251]: This type of actuator technology is emerging as a potential winner for fruit handling. This is due to the use of affordable materials, the simplicity of their manufacture and control, and the grip strength obtained. Special mention should be made of the solution proposed in [223], which can be defined as a hybrid gripper, combining vacuum pressure and an origami-inspired compliant structure. This design has a high gripping force of approximately 50 N, and the authors provide a detailed study of its grasping.
- Tendon-driven [252,253]: This type of technology offers other advantages over the previously mentioned technology, such as greater precision in position control. Specifically, this type of technology can be associated with a structure made up of rigid or soft materials that are passively acted upon by tendons that offer soft-type manipulation.
- FEA-tendon-driven [254]: This approach combines both of the above technologies. Tendon drive technology is used for grasping motions, and actuation is achieved by linear soft vacuum actuators. This type of synergy improves the diversity of objects that can be manipulated, as well as the combination of the advantages of each technology. In one particular case [254], the gripper was able to lift a total of 2.7 kg, which represents a maximum payload-to-weight ratio of 7.06.
- Topology-optimized soft grippers [255]: This type of soft gripper, which operates via elastic deformation, can be adapted to the sizes and shapes of objects without mechanical joints or sensors. In one particular case, the gripper could lift maximum loads of 1.4 kg.

Table 4 gathers the main soft grippers that have been proposed for delicate food handling and robotic harvesting applications. All of them are results of ongoing researches in the field of soft robotics.

As can be seen in Table 4, the cited studies do not list the characteristics of the proposed soft grippers in a homogeneous way, which makes their comparative evaluation difficult. Thus, for example, with regard to the size of the object to be manipulated, each study proposes a different target, which in many cases is carefully selected to ensure an adequate grip. Hence the importance of having standard methods to quantitatively determine and compare the characteristics of soft actuators. It should also be noted that most of the proposed solutions are focused exclusively on the mechanical design, leaving the implementation of the control system for future work. Other crucial aspects such as the adaptation of the grippers to conventional robotic systems, the energy consumption and the power sources required for their operation are not addressed either. More detailed research on the life cycle of actuators is also lacking, which can affect their optimal performance due to the loss of properties that soft materials experience over time.

**Figure 6:**
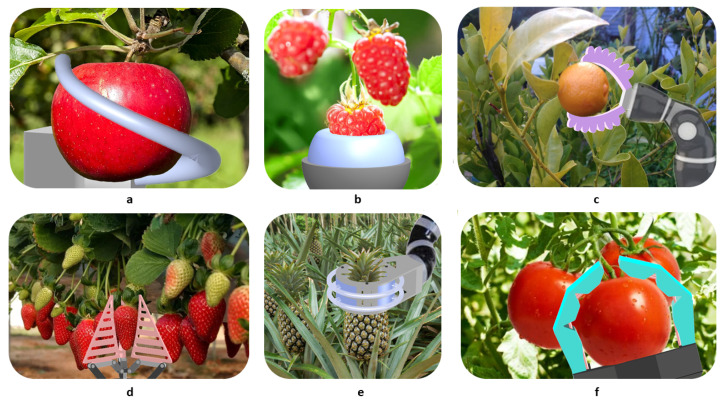
Hypothetical harvest scenarios with several soft grippers. (**a**) Soft continuum gripper based on [256], (**b**) end-effector based on [257], (**c**) bellow-type soft gripper based on [224], (**d**) multi-choice gripper based on [258], (**e**) circular soft gripper based on [220,230], and (**f**) tendon-driven soft gripper based on [254].

Figure 6 displays several soft grippers from the literature that could be adapted for precision harvesting of crops.

### 3.3. Control

Deformability and compliance are some of the main characteristics of soft actuators [259], which translate into a large intrinsic number of DoFs. This obviously affects the control system in terms of complexity. Low-level control for soft actuators, which is highly dependent on the soft materials used, can be decentralized to simplify the complexity [260]. For this reason, it is essential, as a design step, to study the passive mechanical dynamics of soft actuators to achieve the desired deformation behaviour [261].

**Figure 7:**
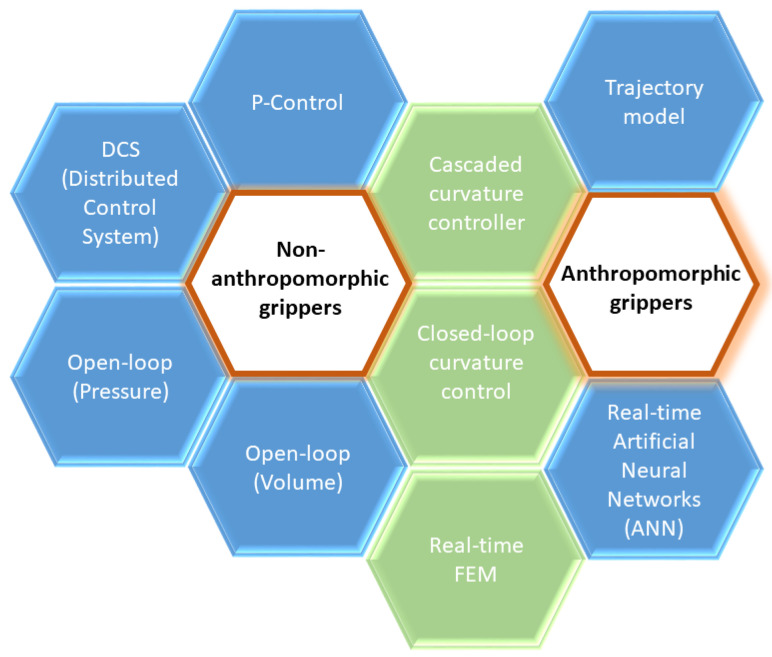
Several control philosophies proposed for FEA-type soft actuators. The control philosophies that have been proposed for a particular type of soft gripper (anthropomorphic or non-anthropomorphic) are presented in blue, while those proposed for both types are shown in green.

As seen above, there are several soft technologies that have their own implications due to the type of actuator they use. Thus, for example, controlling a servo, actuating a cable in tendon-driven technology, controlling compressors and pressure regulators in FEAs, and controlling the amount of electric charge, electro-adhesion, or a thermal stimulus in SMA actuators are different challenges. The geometry of the actuator also has implications for the control system, as it affects the number of axes and movements that soft actuators can execute. The widest variety of control philosophies can be found for FEA soft actuators, as summarized in Figure 7.

Although diverse control strategies have been proposed for FEA-type actuator technology, open-loop control is one of the most frequently used. Several authors [32] report difficulties in controlling certain types of FEA soft actuators due to their deflection around the object. This is especially intricate in anthropomorphic grippers in terms of achieving speed, flexibility and dexterity [191]; not only in FEA actuators but also in passive structures actuated by external motors or tendon motors. This disadvantage can be partially solved by sensing the actuator or by real-time control using FEM [187,262]. On the other hand, tendon-driven soft technology has more mature actuators than pneumatic actuators, and therefore, the control is more straightforward than that of FEAs [237].

## 4. Challenges of Soft Grippers for Robotic Crop Harvesting

Although a number of different soft actuator technologies have been proposed for various applications, soft grippers for robotic crop harvesting are not yet being sufficiently addressed. This is mainly due to the complexity of the unstructured agricultural environment, the intrinsic challenge posed by soft materials and the need to demonstrate the economic viability of robotic harvesting in the sector. Some of the main barriers that soft robotics, and more particularly soft grippers, face against their possible application in agricultural scenarios are listed below.

- Design process: One of the main challenges of soft technology is the design process. A wide diversity of generalist soft grippers can be found in the current state of the art. However, these designs are more focused on achieving new improvements in the field of soft robotics than on developing a specific gripper that solves the issues of the particular field of applications. In terms of robotic crop harvesting, characteristics such as modularity, ease of repair, and the ability to handle food and multiple crops are desired. Apart from this, another gap that needs to be studied is the mathematical model that represents the behaviour of the material in FEM software. This is directly related to the nature of the various materials used, described in the Materials Subsection.
- Repeatability: Another of the main challenges of soft robotics, particular soft grippers, is the need to standardize manufacturing processes. This is the first point to be addressed because it would ensure that the designed soft actuators are suitable for production, facilitating their incorporation into the robotic market. Repeatability studies should research how to mitigate the common effects that appear in soft actuators, such as delamination or interstitial bubbles, that can be the result of faulty manufacturing. To solve these problems, several solutions have been proposed, such as the use of vacuum chambers [227,263,264,265]. Although positive results from this process have been reported, it is impossible to find a method where, for example, variables such as pressure or time are controlled as a function of volume to ensure the repeatability of the process. Obviously, the method would depend on the material used. On the other hand, in most cases, the manufacturing processes are very handmade, and therefore, repeatability can be compromised. However, processes based on 3D printing of soft materials, as well as lost wax manufacturing, may become interesting options in the future, given their greater options for achieving repeatability during the manufacturing process.
- Standard method for determination of soft actuator characteristics: One of the main gaps that has not yet been addressed in soft actuators is the definition of a method to determine their characteristics. However, it is clear that there is a need for a reliable method that can quantify the soft actuator features to facilitate its evaluation and comparison. Properties such as the contact pressure, contact force, contact area and slip force are crucial for benchmarking and determining room for improvement in this field. Thus, this would not only be useful for selecting the optimal option for each process but also for providing a true picture of the progress of this technology.
  In the current state of the art, several approaches can be found for the characterization of soft actuators, in which the experimental measurement is always performed with non-standardized objects. However, the proposed methodologies of the studies differ, presenting various approaches, among which the following stand out.
  – The measurement process proposed in [210] consists of grabbing a spherical object connected by an inextensible cable to a force sensor mounted on a motorized platform to measure the slip properties. A similar approach can be seen in [211] but with a six-axis force transducer.
  – Others, such as [218], use a pressure-mapping sensor to obtain the contact force and the pressure. This method offers a reliable measurement for grasping a static object. Grip strength is measured in a similar way to that in the studies mentioned above.
  – In [230], a payload test is presented to obtain the grip strength. Furthermore, the contact pressure is determined by means of FEM software. This last method can give inaccurate solutions due to its dependence on the mathematical model of the material used.
  – Finally, in [266], a deep and detailed analysis is proposed for the measurement of parameters such as grabbing height, pressure and motion acceleration for a soft actuator. In this case, the tests are carried out not only in static but also in dynamic conditions, differentiating between vertical and horizontal positions. Variables such as size, weight and constituent material are also taken into account, as well as the actuation pressure and the grabbing height. Finally, one of the main contributions of this study is the introduction of the handling ratio, which offers a measurable performance comparison.
- Design of control systems: Most of the soft grippers that have been proposed use open loop control. All of these grippers also have a low-cost goal associated with them. However, this results in impractical soft grippers that are difficult to implement in the agricultural environment. Their lack of real control of the deformation and compliance can affect the handling of fruits in different stages of maturity without damaging them. Thus, the study of new control algorithms that take into account the stiffness of the object to be manipulated is essential for the implementation of soft technology in robotic crop harvesting.
- Improvement of energy source systems: Depending on the type of soft actuator used, the energy support required for the gripper can be an electrical source, a pump or air compressor, or a chemical source. In any of these cases, more efficient equipment must be developed to support these technologies. In the literature, descriptions of energy solutions that drive soft-design systems are scarce. Typically, the proposed solutions are suitable for a laboratory or industrial environment, which is far from the unstructured environments of the agricultural sector. Therefore, the development of new energy solutions must be a compromise between functionality and energy consumption. In addition, the optimisation of the system is necessary not only to increase the autonomy of the overall robotic harvester, but also to simplify it, with the aim of enabling its implementation in current agricultural robotics.
- Economic analysis: Economic studies are often the necessary driving force to incentivize research and development in a given area. In the field of Agriculture 4.0, these economic studies can provide information on the most viable way to harvest different crops. However, at present, there is a need for economic research in this field. A study published in 2019 [267] highlights that only 18 investigations in the literature are dedicated to estimating the profitability of crop automation. This affects not only soft robotics but also other automation technologies, hindering its growth in this sector. However, although the lack of research in this direction is noteworthy, it is clear that in certain crops, such as tomatoes and peppers, the labour cost at harvest time represents 30% of the total cost [268,269]. Thus, mechanical harvesting by using soft grippers may be an economically beneficial alternative to manual harvesting [270].

Another challenge, such as the relatively slower actuation speed, is currently addressed in part with the use of pneumatic channels, also known as pneumatic networks [199] or low-pressure actuators [271]. Furthermore, hybrid gripper technology [272], which combines some advantages of soft and hard robotics, may be another potential solution, providing a soft grip and a structural strength capable of withstanding external agents or objects existing in unstructured environments.

## 5. Conclusions

Agriculture mechanization is still in a growth phase. Tasks such as sowing, weeding and harvesting are the spearhead of the development of Agriculture 4.0. Soft robotics is presented as a suitable technology for the manipulation of fruits and vegetables, which are often delicate and easy to mark or bruise and sometimes slippery. This field of robotics can pave the way for the automation of maintenance, harvesting and post-processing tasks in the agro-food industry.

In this article, a detailed review of the latest advancements in the design of novel soft grippers and end-effectors that could be used for robotic harvesting applications is presented. To that end, the current state of automatic picking tasks for several crops is analysed, identifying the main techniques that are commonly used based on the botanical characteristics of the fruits. Since direct harvesting methods based on twisting, bending, pulling, lifting or a combination of them involve the direct contact with the fruits, the introduction of soft grippers for automation of these techniques may represent a significant advantage, allowing a delicate manipulation that guarantees the integrity of fruits. Direct harvesting techniques with an actuation force on the peduncle are also candidates for the introduction of soft gripper technology, provided they are complemented by a suitable cutting tool.

Regarding the material used for the manufacturing of soft grippers, silicone elastomers are attracting strong interest due to their low cost and because they do not require the use of complex machinery or skilled labour. In addition, these compliant materials are also advantageous when considering the safety of interaction with biological products, making them appropriate candidates for agricultural applications.

It should also be noted that most of the proposed solutions are focused exclusively on the mechanical design, leaving the implementation of the control system for future work. Although diverse control strategies have been proposed for soft actuators, open-loop control is one of the most frequently used. The results of this study also underline that FEA grippers are one of the most promising technologies for robotic harvesting due to their ease of manufacture, compliance and output force. Nevertheless, it is important to note that the implementation of the different soft grippers in agriculture must be associated with the development and improvement in other components of the robotic system, such as artificial vision and navigation.

Furthermore, some of the main challenges that soft grippers still have to overcome to boost is definitive implementation are the design of control systems that consider the stiffness of the fruit to be harvested, the implementation of standardised manufacturing process that guarantee repeatability, the implementation of standard methodologies for the determination of the soft actuators characteristics, and the improvement of the energy sources.

On the other hand, it is important to take into account that the final quality required for fresh market fruits and fruits for the processing industry differs significantly. Soft grippers are presented as the most suitable solution for the harvesting of high value crops, so that mechanical damage is minimised and the products can reach their maximum value in the market. For fruits and vegetables intended for other industrial processing, such as the production of juices, jams and sauces, the economic feasibility of solutions based on soft grippers should be further evaluated. Therefore, future research should be directed to conducting economic studies that provide information on the most viable way to harvest different crops [267], and on the measures that should be taken to minimize losses [273]. Moreover, the study of methods to accurately assess the extent of surface and internal fruit damage caused by excessive external forces should also be addressed [274]. Finally, it would be convenient to carry out studies that analyse the life cycle of soft actuators made with silicone elastomers, to determine if their degradation may leave particles on the products manipulated.

## Figures and Tables

**Table 1:** Classification of existing grippers.

Type	Bending Characteristics	Shearing Characteristics
Peduncle rotation [61]	X	-
Pushing some object into peduncle [53]	X	-
Knife, one sided blade [53,77]	-	X
Scissors [78]	-	X
Saw [49,53]	-	X
Hot wire [54,79]	-	X

**Table 2:** Simple fleshy fruit classification.

Type of Fruit	Name	Actual Harvesting Method	Automatic Harvesting Method
Drupes	Apricot	2	1 [84,85]
	Blackberry	2	1 [86]
	Cafe	2	1 [87,88]
	Cherry	2	1 [89,90,91,92,93,94,95], 2 [96], * [97]
	Coconut	3	3 [98,99]
	Loquats	2	-
	Lychee	3	* [100,101,102,103]
	Mango	2,3	1 [104], 2 [105], 3 [106,107,108], * [109]
	Nectarine	2	* [110]
	Olive	1	1 [111,112,113,114,115]
	Peach	2	1 [116], * [117,118]
	Plum	2	2 [119]
	Raspberry	2	1 [120,121]
Berries	Avocado	3	* [122]
	Blueberry	2	1 [123]
	Eggplant	3	3 [50,51], * [124]
	Grape	1,3	1 [125,126,127,128], 2 [129], * [130]
	Guava	3	* [131]
	Kiwi	2	2 [132,133,134]
	Papaya	2	3 [105], * [135]
	Passion fruit	2	* [136]
	Pepper	3	3 [55,56,57,137,138,139], * [140,141]
	Persimmon	2	2 [142], * [143]
	Pitaya	3	* [144]
	Pomegranate	3	* [145]
	Tomatoes	2,3	2 [46,47,48,49], 3 [146,147], * [148,149,150]
	Wolfberry	2	1 [151], 2 [152], * [153]
Pomes	Apple	1,2	1 [154], 2 [42,43,44,45,155,156], 3 [157], * [158,159]
	Pear	2	3 [157], * [160]
	Quince	2	-
Hesperidium and Pepo	Banana	3	* [161]
	Cucumber	3,2	3 [54], * [162,163]
	Grapefruit	3	1 [164]
	Lemon	3	* [165]
	Lime	3	-
	Melon	3	3 [52]
	Orange	3	1 [34,164,166,167], 3 [53], * [168,169]
	Pumpkin	3	2 [170], * [171]
	Watermelon	3	3 [172]

(*) Artificial vision research.

**Table 3:** Aggregate and multiple fruit classification.

Type of Fruit	Name	Actual Harvesting Method	Automatic Harvesting Method
Aggregate fruit	Custard Apple	2	-
	Strawberry	2	2 [40,41,66,67,173,174], * [175,176]
Multiple fruit	Fig	2	-
	Pineapple	2	2 [177], 3 [178], * [179,180,181]

(*) Artificial vision research.

**Table 4:** Literature review of food soft grippers.

Soft Technology	Reference	Grasped Object	Object Size or Weight	Gripper Type	Gripper Size	Lifting Ratio	Scalability	Controllability	Response Time	Surface Condition	Mechanical Compliance	Lifetime
FEAs	[251] *	Lettuce	250 × 250 mm	Two pneumatic actuators and a blade	8000 g, 450 × 450 × 300 mm	-		Close-loop with force sensor feedback	31.7 s	-		-
	[236] *	Apple	-	Three soft finger design	Two fingers length: 95,25 mm One Finger length: 152,4 mm	-	-	Open-loop	7.3 s	-		-
	[230]	Mushroom	-	Three soft chambers in circular shell	Chamber height: 20 mm Chamber arc angle: $60^{{}^{\circ}}$	30		-	-	Any surface	-	-
	[246]	Apple, Tomato, Carrot, Strawberry	69 mm, 5–150 g	Magnetorheological gripper	-	-	-	PID	0.46 s	Any surface		-
	[248]	Cupcake liners filled with peanuts	34–64 g	Three soft finger design	Finger size: 82 × 16 × 15 mm	-		FE analysis	-	-		-
	[250]	Cupcake liners filled with red beans, higiki, ohitashi	75.2 g	Soft fingers	Finger length: 97 mm	1805%		Open-loop	10 s pick and place (total procedure)	-	-	1100
	[224]	Defrosted broccoli	33.54 × 23.94 mm, 3.8–7.0 g	Two soft fingers	Actuator size: 50 × 20 mm	-	-	-	3 s for inflation
	[225]	Granular kernel corn, Chopped green onion, Boiled hijiki	0.77–26.6 g	Four soft fingers	Finger size: 43 × 61,5 mm	-		Open-loop	-	Any surface	-	-
	[206]	Orange	1000 g	Soft fingers	Finger size: 95 × 20 × 18 mm	-		Open-loop	-	Any surface	-	-
	[220]	Tomato, Kiwifruit, Strawberry	45 to 76 mm	Four soft chambers in circular shell	Internal diameter: 46 mm Height: 30 mm	-		Open-loop	2–5 s	Any surface		-
Tendon-driven	[253]	Tomato	500 g	Three soft finger design	-	-		Preprogrammed rotation of motors	-	-		1000
	[252]	Tomato, Cucumber (slices) Avocado (Strips) Cherry Tomato, Olives, Pineapples cubes, Broccoli	-	Quad-Spatula design	-	-		-	-	Flat surfaces	-	-
FEA-Tendon-driven	[254]	Banana, Apple, Grapes	2700 g	Three soft finger design with a suction cup	389.69 g	7.06		Teleoperation Control	0.094 s (Rise time)	Any surface (irregular shapes and sharp corners)		26–20 cycles
Topology optimized soft actuators	[255]	Apple, Grapefruit, Guava, Orange, Kiwifruit	1400 g	Two compliant fingers	-	-		Open-loop (Arduino)	-	-		-

* Soft Gripper for harvesting purposes.-Data not provided.
